# Adaptive Data Filtering of Inertial Sensors with Variable Bandwidth

**DOI:** 10.3390/s150203282

**Published:** 2014-02-02

**Authors:** Mushfiqul Alam, Jan Rohac

**Affiliations:** Department of Measurement, Faculty of Electrical Engineering, Czech Technical University in Prague, Technicka 2, Prague 16627, Czech Republic; E-Mail: jan.rohac@fel.cvut.cz

**Keywords:** inertial navigation, attitude control, filtering algorithms, adaptive signal processing, accelerometers

## Abstract

MEMS (micro-electro-mechanical system)-based inertial sensors, *i.e.*, accelerometers and angular rate sensors, are commonly used as a cost-effective solution for the purposes of navigation in a broad spectrum of terrestrial and aerospace applications. These tri-axial inertial sensors form an inertial measurement unit (IMU), which is a core unit of navigation systems. Even if MEMS sensors have an advantage in their size, cost, weight and power consumption, they suffer from bias instability, noisy output and insufficient resolution. Furthermore, the sensor's behavior can be significantly affected by strong vibration when it operates in harsh environments. All of these constitute conditions require treatment through data processing. As long as the navigation solution is primarily based on using only inertial data, this paper proposes a novel concept in adaptive data pre-processing by using a variable bandwidth filtering. This approach utilizes sinusoidal estimation to continuously adapt the filtering bandwidth of the accelerometer's data in order to reduce the effects of vibration and sensor noise before attitude estimation is processed. Low frequency vibration generally limits the conditions under which the accelerometers can be used to aid the attitude estimation process, which is primarily based on angular rate data and, thus, decreases its accuracy. In contrast, the proposed pre-processing technique enables using accelerometers as an aiding source by effective data smoothing, even when they are affected by low frequency vibration. Verification of the proposed concept is performed on simulation and real-flight data obtained on an ultra-light aircraft. The results of both types of experiments confirm the suitability of the concept for inertial data pre-processing.

## 1. Introduction

Recently, there has been a growing trend toward using cost-effective MEMS (micro-electro-mechanical system) technology-based sensors for navigation purposes in aerospace systems, such as on light aircrafts and unmanned aerial vehicles (UAVs). A strapdown inertial system consisting of tri-axial accelerometers (ACCs) and tri-axial angular rate sensors (ARSs) is commonly used for attitude estimation (roll, pitch, yaw angle), as well as for velocity and position evaluations. On large aircraft, ring laser gyros and servo ACCs are used on board for precise measurements, which is expensive. In comparison, MEMS sensors are compact, lightweight and cost effective, thus offering an inexpensive solution for navigation purposes. However, at the same time, MEMS-based inertial sensors suffer from bias instability, insufficient sensitivity, noise, *etc.*, which present significant challenges in data processing that have to be dealt with in navigation processes. Originally, the attitude was supposed to be evaluated by integrating angular rates; nevertheless, as mentioned before, the measurements suffer from several inaccuracy impacts. In the case of the ARS-based attitude evaluation process, this inaccuracy causes unbound error growth, which needs to be corrected by data obtained from so-called aiding systems, e.g., magnetometers, cameras and even ACCs. These aiding systems provide information about attitude only under certain conditions, limiting their usability. This paper focuses on data pre-processing for navigation solutions based on inertial sensors only (ARSs and ACCs). Therefore, the ARS-based attitude evaluation process is primarily aided by ACC-based attitude evaluation [1]. This aiding can be applied under conditions when only gravity affects ACC measurements and no other acceleration is present [2–4]. This principle is common in cost-effective solutions of attitude and heading reference systems (AHRSs); however, these ideal aiding conditions are hardly achievable in harsh environments, due to strong vibrations present on light or small aircrafts. This complicates the situation, as the frequency of those vibrations cannot be simply isolated from the aircraft dynamics. The ARSs are primarily used for attitude evaluation, unlike the ACCs, which are utilized in AHRS just for attitude compensation.

**Figure 1.**
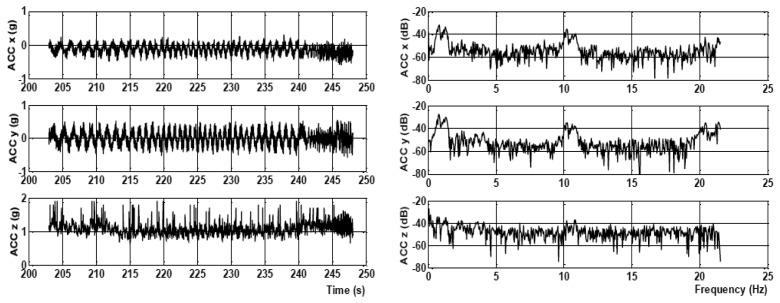
Accelerometer (ACC) measured during engine suppression and the frequency spectrum.

**Figure 2.**
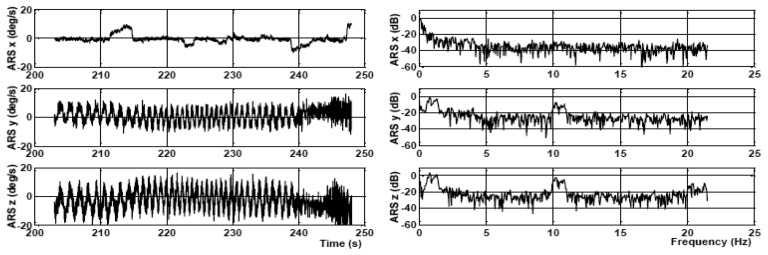
Angular rate sensor (ARS) measured during engine suppression and the frequency spectrum.

To learn the characteristics of real flight conditions, several flight experiments were performed using IMU ADIS16350 (Analog Devices, Norwood, MA, USA), which was utilized in the EFIS INTEGRA TL-6524 (Electronic Flight Instrumentation System) ,flight monitoring system manufactured by TL-Elektronic, Inc. (Hradec Králové, Czech Republic) The system was mounted to the instrument panel of the ATEC321 aircraft (ATEC321 is a Czech ultra-light aircraft, designed and produced by ATEC v.o.s, Libice nad Cidlinou, Czech Republic). The instrument panel was equipped neither with active nor passive vibration dampers. As a result, the sensors were directly affected by strong structural vibrations. Measurements were made for different flight phases, such as parking, taxing on the runway, taking off, during the flight and landing. The data were recorded at a sampling frequency of 43 Hz. The worst situation corresponds to the case when the vibration impact cannot be distinguished and isolated from the aircraft dynamics. Such a situation is depicted in Figures 1 and 2, which show the flight data from ACC and ARS measured during the engine revolution per minute (RPM) suppression and their frequency spectrum.

In cases of engine RPM suppression during the flight, vibration frequencies go down all the way to 0.5 Hz. ACCs are generally affected by the combination of translation, centrifugal and gravitational accelerations along with the vibrations arising from the propeller and the aircraft structure. The vibration effect often dominates the ACC measurements. In contrast, vibrations have slight impacts on ARS readings depending on *g* and *g^2^*sensitive parameters; contrariwise, their readings are affected by bias instability and noise. These different characteristics of ARS and ACC enable their data fusion to improve the final accuracy of the whole attitude estimation process. Generally, ARS data are always used to estimate attitude, even under dynamic conditions, when the aircraft is maneuvering or under steady flight conditions. Unlike ACC, data are directly utilized for the attitude compensation under only steady-state conditions when the gravity distribution in the sensor's framework can be estimated. This corresponds to situations in which the aircraft performs a direct and unaccelerated flight. In the cases where the aircraft undergoes a banked turn or a circular flight, a long-term additional acceleration is present due to the centripetal force created by traveling along a curved path. In such conditions, it is possible to estimate the centripetal acceleration and to subtract it out by providing the known velocity or airspeed to the data fusion process [5]. There exist several approaches to data fusion for attitude estimation, such as temporally-interconnected observers (TIO) [6], complementary filters [7] or Kalman filters [8,9]. However, the accuracy of the estimation is always reduced, while the ACC data are affected by periodic vibration. This complicates the situation, due to a harsh environment causing structural vibrations, which are directly picked up by the ACCs. Therefore, for precise attitude estimation regardless of the aircraft flight condition, it is essential to provide acceleration data that are as smooth as possible with a reduced vibration effect; thus, ACC data require pre-processing. The light aircrafts are classified as Level I Class I, Category B Flight Phase (cruise, climb, descent, loiter) by the Federal Aviation Administration (FAA). The maximum time to achieve a change in the bank and pitch angle is 1.7 s, and the minimum time is 0.2 s [10,11]. This means that the aircraft's operational frequency lies in the range of 0.6 up to 5 Hz. In this instance, the ideal choice would be to apply a band-pass (BP) filter; nevertheless, such a narrow bandwidth would require a very high order filter, which is not desirable for navigation purposes, because of the long delays.

As mentioned, the aircraft dynamics lies in the range of 0.6 up to 5 Hz, and the vibration frequency might go all the way down to 0.5 Hz. Therefore, using a constant 5 Hz bandwidth low-pass (LP) filter would mean that the 0.5 Hz frequency vibrations will not be filtered. On the other hand, using a constant 0.6-Hz bandwidth LP filter would lead to a situation in which the aircraft's motion information in the bandwidth up to 5 Hz would be lost. Therefore, our contribution is a novel concept of pre-processing ACC data using adaptive bandwidth filtering, which is modified based on sinusoidal data estimation. The proposed filtering algorithm is adaptive in the sense that the filtering bandwidth is modified based on the signal history. This enables the usage of ACC-based attitude compensation, even under variable low-frequency vibration impacts, while common commercial AHRS systems fail to have the correct compensation capability. This proposed approach brings several advantages against the ones commonly used, such as a smaller and acceptable delay, even when the narrowest bandwidth of 0.5 Hz is applied on the ACC data. On the other hand, ARS data are filtered with a constant bandwidth, and thus, when no low-frequency vibrations arise, all data are filtered with the same bandwidth of 5 Hz, which provides the same delay of data pre-processing for the majority of the flight. This approach hence brings an added advantage to inertial data pre-processing and enhances the ACC-based attitude compensation possibilities.

The rest of the paper is organized as follows: Section 2 outlines the methodology of the proposed concept in detail. A detailed description of the principle of estimating the sensor's signal via a sinusoidal estimation filtering algorithm is also presented in this section. Section 3 provides the results of the proposed algorithm applied on simulated data and real flight data and confirms the suitability of the approach. Section 4 concludes the paper with final remarks.

## 2. Methodology

This paper proposes an adaptive variable bandwidth filtering via sinusoidal data estimation to pre-process the data of ACCs and ARSs by as narrow a bandwidth LP filter as possible, while preserving the dynamics information included in the data. In the past, several attempts were made to use variable bandwidth filtering in communications systems [12,13], but the use was limited to a fixed length of finite impulse response (FIR) filters. However, the length of the filter is always proportional to its delay, which restricts the usability for navigation purposes. In the proposed concept, two key assumptions are made:

1. The vibration content in the inertial data has an approximately periodic and sinusoidal characteristic.
2. The frequency of the signal content varies gradually, and the changes are smooth.

**Figure 3.**
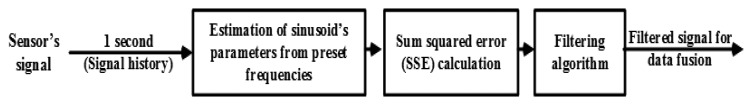
Complete filtering block diagram.

A schematic block diagram of the proposed signal filtering method using variable bandwidth filters is depicted in Figure 3.

In general, the overall filtering process can be broken down into three main stages: (1) estimation of the sinusoid's parameter; (2) sum squared error calculation; and (3) applying filtering on the signal. The overall filtering task is carried out with a 1 s window of the signal history. This particular length of the history is chosen to have the capability to detect vibration content down to 0.5 Hz, since at least a half cycle of the approximate sinusoidal signal is necessary for the estimation process. The estimation of the sinusoid's frequency is based on preset frequencies, which are chosen according to the required filtering pass bands and bandwidths associated with the flight operational conditions. When raw signals enter the first block, they are fitted with sinusoids of all preset frequencies to get their approximations. The raw signals and their sinusoidal approximations are then led to the second block to calculate the sum squared error (SSE) of the fitting. The sinusoidal approximation with the preset frequency for which the SSE is the lowest marks the best fit and, thus, indicates the operating frequency of the vibration's strongest content. Finally, based on the operating frequency, a variable bandwidth in the filtering algorithm is adapted and applied on the signal. The process in the first and second block can be easily performed by fast Fourier transformation (FFT) while post-processing; however, it is computationally expensive for real-time applications. Thus, applying the proposed sinusoidal approximation technique brings an advantage in terms of lower computational demands, making the filtering suitable for real-time applications. Details about the chain of signal processing described above are presented in the following subsections.

### 2.1. Principles of Sinusoidal Signal Estimation

As mentioned earlier in Section 1, the vibration impact on the sensor's signal is often periodic, sinusoidal in nature and with one strongest frequency content. Therefore, it is often a reasonable approximation to address the problem of determining/estimating the frequency content in the raw signal via sinusoidal fitting algorithms.

Assume that the signal history *x* is obtained at time instances *t* where *N* is the total number of samples in the sequence. *N* is chosen to preserve a 1 s window. *x_N_* and *t_N_* correspond to the latest sample, and (*N* − 1) down to 1 represent samples in the signal history.

(1) $$x_{n}={\left[x_{1}\;x_{2}\;\ldots x_{N-1}\;x_{N}\right]}^{T};\;t_{n}=\left[t_{1}\;t_{2}\;\ldots t_{N-1}\;t_{N}\right]$$

For a small signal history window, the history sequence *x* can be assumed as periodic with a frequency *f* and angular frequency *ω* = 2*πf*. The orthogonality relationships of the sine and cosine functions can be used to break down an arbitrary periodic function into a set of simple terms that can be summed, solved individually and then recombined to obtain the solution to the original signal sequence *x_n_* or to its approximation. Using the method for a generalized Fourier series, the signal history sequence *x* can be represented or modeled as a summation of the cosine and sine, as follows:

(2) $${\text{s}}_{n}\left(\vartheta \right)=A\;\text{cos}\left(\omega \times t_{n}\right)+B\;\sin\left(\omega \times t_{n}\right)+C$$

where *s_n_* is the approximation of *x_n_*; *A*, *B*, *C*, *ω* and *f* are unknown constants, and *ϑ* defines the set of these four unknown parameters (*A*, *B*, *C* and *ω*). The sine wave fitting problem in (1) and (2) can be then solved by minimizing of the sum squared error [14,15], which is given by:

(3) $$V\left(\vartheta \right)=\frac{1}{N}\overset{N}{\underset{n=1}{\text{∑}}}{\left(x_{n}-s_{n}\left(\vartheta \right)\right)}^{2}$$

Consider the particular parameter vector *θ*; where *θ* = [*A B C*]*^T^* and *ϑ* can be written as:

$$\vartheta ={\left[\theta^{T}\omega \right]}^{T}$$

Let *D* (*ω*) be the *N* × 3 matrix defined as:

(4) $$D\left(\omega \right)=\left[\begin{array}{ccc} \text{cos}\left(\omega \times t_{1}\right) & \text{sin}\left(\omega \times t_{1}\right) & 1\\ \vdots & \vdots & \vdots \\ \text{cos}\left(\omega \times t_{N}\right) & \text{sin}\left(\omega \times t_{N}\right) & 1 \end{array}\right]$$

The sum squared error in (4) can be written as:

(5) $$V\left(\vartheta \right)=V\left(\omega ,\theta \right)=\frac{1}{N}\left\{{\left[x_{n}-D\left(\omega \right)\theta \right]}^{T}\left[\left(x_{n}-D\left(\omega \right)\theta \right)\right]\right\}$$

When the frequency *f* of the signal history is known (in other words, angular frequency *ω* is known), Equation (5) can be minimized in the least squares sense by solving the set of linear equations *D*(*ω*) *θ* = *x_n_* [16]. If *D*(*ω*) has the full rank, the solution of the estimated *θ̂* is given by:

(6) $$\hat{\theta }=\left(\begin{array}{c} \hat{A}\\ \overset{⌢}{B}\\ \hat{C} \end{array}\right)={\left(D{\left(\omega \right)}^{T}D\left(\omega \right)\right)}^{-1}D{\left(\omega \right)}^{T}x_{n}$$

It can be noted that for large *N*, the columns in *D*(*ω*) become orthogonal. Thus, *D*(*ω*)*^T^ D*(*ω*) becomes a diagonal matrix with elements $\left[\overset{N}{\;}/\underset{2}{\;}\;\overset{N}{\;}/\underset{2}{\;}\;N\right]$. Thus, it makes the calculation of the inverse of (*D*(*ω*)*^T^ D*(*ω*)) to estimate *θ̂* computationally inexpensive.

### 2.2. Sum Squared Error Calculation to Estimate the Filtering Bandwidth

The principles described in Section 2.1 are used to estimate the frequency content in the signal considering the 1 s window of the signal history. The mentioned Equations (5) and (6) can be solved easily when the frequency *f* is known. Therefore, the proposed approach uses preset frequencies *f̂_i_*. These frequencies specify the filtering bandwidth, which can be then applied in the third block in Figure 3. Each *f̂_i_* is used to estimate *D*(*ω*) defined in Equation (4) and *θ̂* in Equation (6). The sinusoidal signal *x̂_ι_i__* is then constructed for each *f̂_i_* and the corresponding *θ̂* using:

(7) $$\hat{x_{n_{i}}}=\hat{A}\;\text{cos}\left(2\pi {\hat{f}}_{i}\times t_{n}\right)+\hat{B}\;\text{sin}\left(2\pi {\hat{f}}_{i}\times t_{n}\right)+\hat{C}$$

This step gives the advantage of using as many estimates as needed for the specific application and required filtering bandwidths. To get the best sinusoidal approximation with respect to the original raw signal *x_n_*, the *SSE* is calculated using:

(8) $$\text{SSE}=\overset{N}{\underset{1}{\text{∑}}}{\left(x_{n}-\hat{x_{n_{i}}}\right)}^{2}$$

The preset frequency *f̂_i_*, which gives the lowest value of the SSE, provides the best fit for the measured signal and, thus, indicates the operating frequency based on which the filtering bandwidth of the third stage is adapted and applied. Since the estimation process uses the 1 s window, the proposed approach has a corresponding learning time of 1 s. This means that the proposed approach will take 1 s to detect a complete change/transition in the frequency and to adapt the filtering bandwidth.

### 2.3. Filtering Algorithm

**Figure 4.**
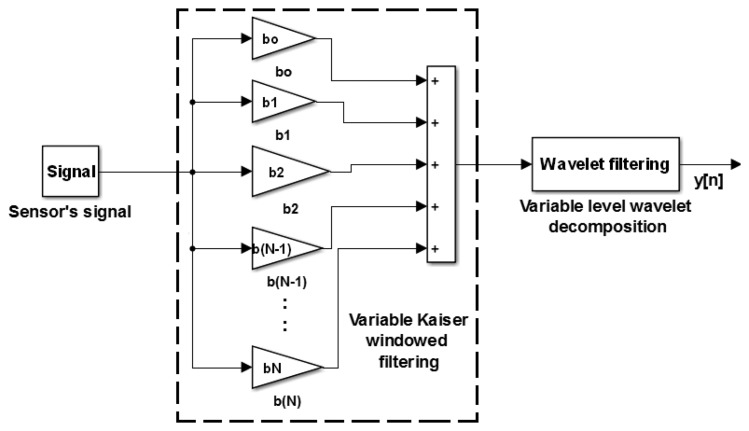
Schematic diagram of the new filtering algorithm.

A conventional finite impulse response (FIR) filtering (such as *generalized equiripple*, quadratically weighted moving average, *etc.*) alone cannot be used for such a low filtering bandwidth (≈0.5 Hz) while providing smooth data. In addition, reaching such a narrow bandwidth would lead to higher order filters, which is not desirable, since they produce a long delay in the signal processing. Therefore, a novel multistage adaptive filtering approach is developed, as demonstrated in the block scheme in Figure 4. The proposed filtering process is adaptive in the sense that the bandwidth of the overall filtering process can vary with respect to the frequency content in the signal. The filtering process can be broken down into two main stages. The first stage is the filtering of the signal using a variable bandwidth Kaiser windowed filter with coefficients [*b*_1_ *b*_2_… ‥ *b_N_*_−1_ *b_N_*], while the second stage utilizes an LP wavelet filter with a variable level of decomposition.

For the first stage, a Kaiser windowed LP filter is chosen, since it allows to control the transition band, pass band and stop band ripples through a proper choice of the filter order and has further a unique *sin* function shape, which provides low bandwidth and low side lobes at an equivalent filter length compared to other types of conventional filters. The coefficients of the Kaiser windowed LP filter can be calculated as:

(9) $$b\left(n\right)=\frac{I_{o}(\frac{2\beta }{M}\left(\sqrt{n\left(M-1\right)}\right)}{I_{o}\left(\beta \right)}$$

where *β* is an arbitrary, non-negative real number that determines the shape of the window, *I_o_* is the zeroth order modified Bessel function and *M* is the order of the filter.

The second stage is formed by a wavelet filter, which brings further advantages in terms of a further attenuation of high frequency noise, while preserving an acceptable delay. Different levels of wavelet decomposition can be reached by taking an LP mother wavelet filter, upsampled by a factor of 2 and convolving it with the same LP mother wavelet filter. This process can be repeated to achieve different levels of decomposition and, thus, different filtering bandwidths. In our case, the *sym*4 mother LP wavelet was considered. The details of the choice of mother wavelet filter and for obtaining different levels of decomposition are outlined in [17–20].

The filtering bandwidth of wavelet filtering cannot be explicitly chosen or controlled; however, wavelet filters are capable of providing smooth data for signal reconstruction. Whereas the filtering bandwidth for the Kaiser window can be chosen based on Equation (9), the two characteristics of the two filters can be combined together to provide one overall filter that is efficient in low frequency attenuation, while keeping the filter order minimal; in other words, keeping the delay minimal.

In the filtering process, the raw signal is passed through the Kaiser windowed LP filter and then filtered by the wavelet filter to further suppress the high frequency noise and to smooth the filtered signal. Note that the overall filtering bandwidth can be modified by varying the length of the Kaiser windowed LP filter and by modifying the level of the wavelet filter decomposition. Simulation results are discussed in detail in Section 3.2, and the experimental verification is in Section 3.4.

## 3. Performance Evaluation and Discussion

The performance of the proposed filtering approach is evaluated based on simulated data, as well as on real flight data. The algorithm was implemented using MATLAB. The results are presented in detail in the following subsections.

### 3.1. Performance of the Filtering Algorithm with Different Bandwidths

As mentioned above, a main objective of the proposed filtering algorithm is to achieve efficient filtering performance in terms of low frequency vibration attenuation in the signal while preserving an acceptable delay. For this reason, we have split the frequency range of interest, 0.5 to 5.5 Hz, into 11 bandwidths with a step size of 0.5 Hz. Based on the required bandwidth values, we have optimized the coefficients of the Kaiser windowed LP filter using Equation (9) and chose the levels of wavelet filter decomposition.

**Figure 5.**
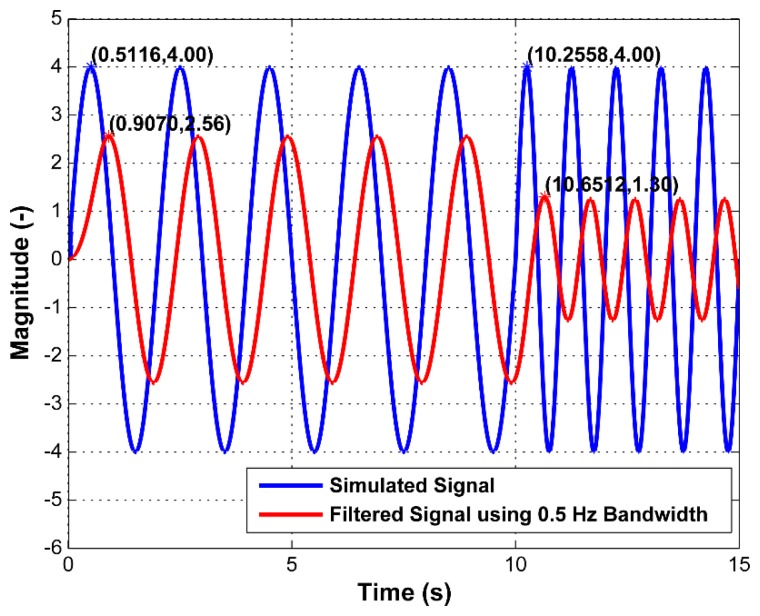
Filtering the simulated signal using only the 0.5 Hz bandwidth filter.

To observe the efficiency of the overall filtering, we simulated a sinusoidal signal with frequencies in the range of 0.5 to 14 Hz and let it pass through both stages of the proposed filtering algorithm. The sampling frequency of the simulated signals was chosen to be 43 Hz to be consistent with the sampling frequency of the real flight experiment. The performance of the filtering for two signal frequencies (0.5 Hz and 1 Hz) when the bandwidth was set to 0.5 Hz is depicted in Figure 5. For all combinations of the bandwidths and the signal frequencies, see Table 1, which summarizes the filtering performance. Particular delays corresponding to the filtering performances are denoted in Table 2. It can be seen that the maximum delay in the signal is about 0.39 s, which is an acceptable delay for ACC signal processing and attitude compensation.

The attenuation level in the filtered signal is calculated using:

(10) $$G_{\text{dB}}=20\log_{10}\left(A_{2}/A_{1}\right)$$

where *A*_1_ is the amplitude of the original signal and *A*_2_ is the amplitude of the filtered signal.

The selection of the filtering bandwidth that is applied in the third block in Figure 4 is dependent on Tables 1 and 2. In Table 1, the minimum required level of attenuation corresponds to −15 dB; nevertheless, if further attenuation is needed, it is always a possibility to use a narrower bandwidth up to 0.5 Hz. Attenuation of −15 dB corresponds to approximately 1/5th of the original amplitude. The particular choice of filtering bandwidth based on the signal operating frequency is highlighted in dark grey in Table 1. For example, if the operating signal frequency is 10 Hz or higher, the filtering bandwidth is 5.5 Hz. If it is from 8 to 10 Hz, the filtering bandwidth is going to be 5 Hz.

### 3.2. Demonstration of Data Smoothing

**Figure 6.**
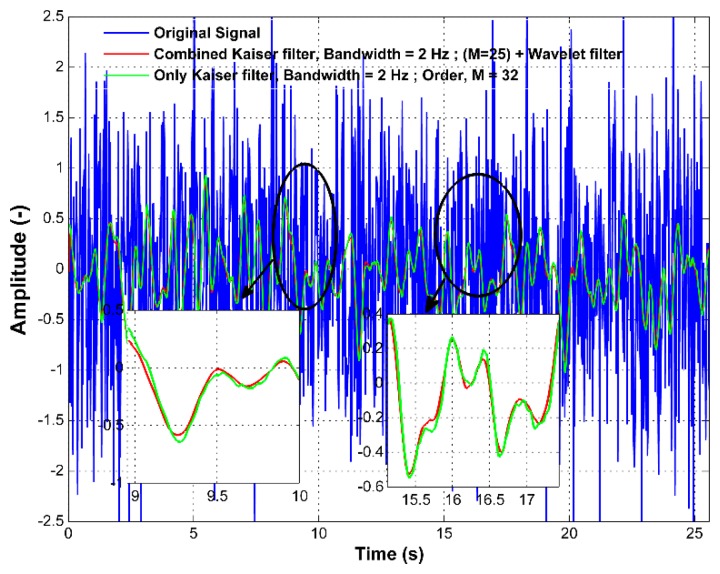
Comparison between filtering using only the Kaiser windowed low-pass (LP) filter and the proposed filtering multistage algorithm with the wavelet filter implemented in the time domain.

**Figure 7.**
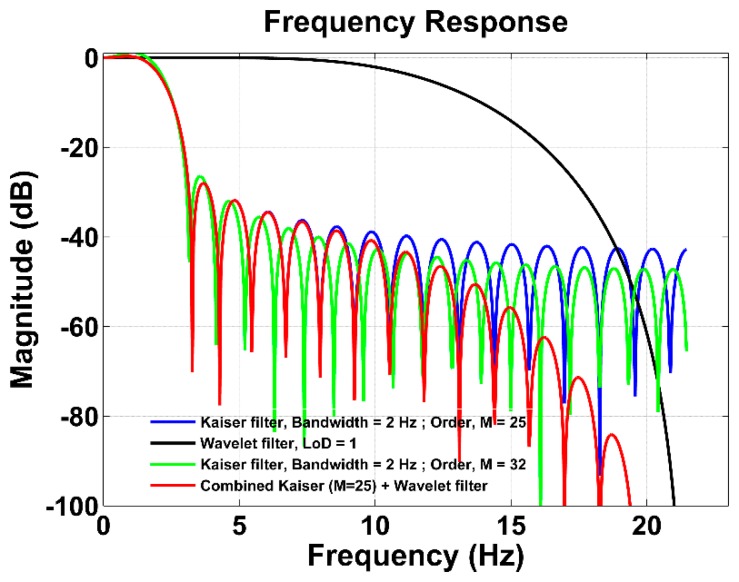
Comparison between filtering using only the Kaiser windowed LP filter and the proposed filtering multistage algorithm with the wavelet filter implemented in the frequency domain.

As mentioned in Section 2.3, the wavelet filters are used to smooth the signal, as well as to further attenuate its unwanted high frequency content. To confirm these characteristics, a white noise was generated with variance set to unity. Two LP Kaiser filters were designed for the bandwidth of 2 Hz with different filter orders (M = 25 and M = 32) using Equation (10) and applied to the white noise. Afterwards, the signal filtered with the Kaiser filter of M = 25 was further passed through the wavelet filter with level of decomposition (LoD) = 1. The filtering results are shown in Figure 6. The results are further demonstrated in the frequency domain in Figure 7.

In Figure 7, it can be seen that both LP Kaiser filters with a cut-off frequency of 2 Hz have slightly different attenuation at higher frequencies; however, their delays are different, *i.e.*, 0.28 s and 0.36 s. Nevertheless, when the LP Kaiser filter of M = 25 (blue line) is combined with the wavelet filter with the first level of decomposition (LoD = 1) (black line), which has M = 7, the combined filter performance (red line) changes for the higher frequencies, while preserving the attenuation at low frequencies. The overall filtering has the order M = 32, and its filtering efficiency increases when attenuating higher frequencies. Thus, it reaches better performance than using only the LP Kaiser filter of M = 32 with a comparable time delay. This means that the proposed filtering approach provides a more enhanced filtering capability compared to conventional filters, while keeping the filter order at a minimum.

This can be explained by the wavelet filters being advantageous despite having an irregular shape to their frequency characteristics. They are able to perfectly reconstruct functions with linear and higher order polynomial shapes, such as rectangular, triangular, second order polynomials and windowed filters [21]. Note that Fourier series fail to do so while designing regular filters, such as Kaiser, and various other conventional filters mentioned earlier [22]. As a result, wavelets are able to denoise the particular signals better than conventional filters that are based on the Fourier transform design and that do not follow the algebraic rules obeyed by the wavelets.

### 3.3. Application of the Multistage Filtering Algorithm on Simulated Data

**Figure 8.**
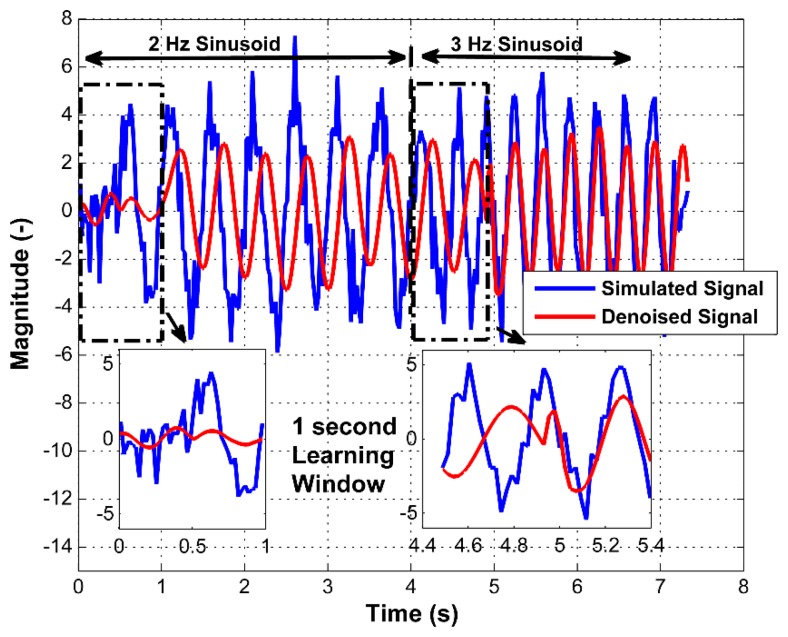
Filtering result on the simulated signal.

For testing the adaptability of the proposed filtering approach, a sinusoidal signal was simulated with two frequencies (2 Hz and 3 Hz) with additive white Gaussian noise of unity variance and a sampling frequency of 43 Hz. It was observed how the filter behaves with respect to a change of frequency. The resulting performance is shown in Figure 8.

The first part of Figure 8 contains the signal with 2 Hz, while 3 Hz is used in the second part. From the filtered data, it can be seen that the filtering method has a 1 s learning time to observe the complete transition between one operating frequency to another. The figure also illustrates that for a low frequency (2 Hz), a lower filtering bandwidth is used than in the case of a frequency of 3 Hz. The zoomed part of the time range of 4.4 to 5.4 s shows the filter behavior when the filtering bandwidth is changed.

### 3.4. Application of the Algorithm on Real Flight Data

As mentioned in Section 1, a narrow bandwidth filtering is required due to the potential presence of low frequency vibrations affecting inertial sensors. Aircraft can fly under direct and un-accelerated conditions or under dynamic motion conditions. For cost-effective attitude estimation systems, it is necessary to consider that the signals from the ARSs require preserving the dynamics information, and in contrast, the ACCs' signals are used as an aiding source to compensate for the attitude estimation, only under steady flight conditions. Mentioned in Section 1, the motion dynamics of light aircraft lies within the 5 Hz bandwidth; hence, a constant bandwidth of 5.5 Hz is used for the ARSs' signal filtering. This choice provides unmodified dynamics in the range of 5 Hz, as required. In the case of ACC signals, an adaptive bandwidth filtering is applied to reduce the vibration effects in the signals. In other words, the same bandwidth of 5.5 Hz is chosen for both ARSs and ACCs when the flight conditions lead to noise and vibrations with frequencies higher than 10 Hz. This ensures the same delay for both ARSs and ACCs for the majority of the flight, which is advantageous. When conditions change and the vibration frequency goes down, the ACCs' signal filtering bandwidth is adapted accordingly, potentially down to 0.5 Hz, while the filtering bandwidth for the ARSs' signals does not change. This approach provides both observable dynamics from ARSs and a compensation capability for attitude estimation with the help of the ACCs, even under steady flight conditions when the ACCs are affected by low frequency vibrations. Generally, due to the low frequency vibrations' influence, the operation of the majority of the commercially used cost-effective AHRSs is limited. In contrast, the proposed filtering approach is more robust while operating under a low frequency vibrating environment compared to the commonly used approaches.

**Figure 9.**
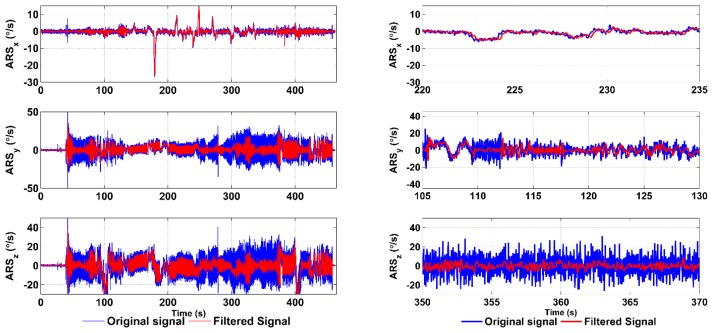
Angular rates measured during the flight, the filtered signals and the zoomed tracks.

Real flight data for ACCs and ARSs are obtained from flight experiments using the ultra-light aircraft, ATEC 321. The flight data were sampled at a frequency of 43 Hz. As proposed, for the filtering purposes, 11 different bandwidths were chosen at equal intervals from 0.5 Hz to 5.5 Hz. The filtering of the ARS data using a constant 5.5-Hz bandwidth LP filter is shown in Figure 9. It can be seen that the potential high frequency noise is attenuated and filtered in the ARS measurements, while preserving the delay up to 0.23 s.

**Figure 10.**
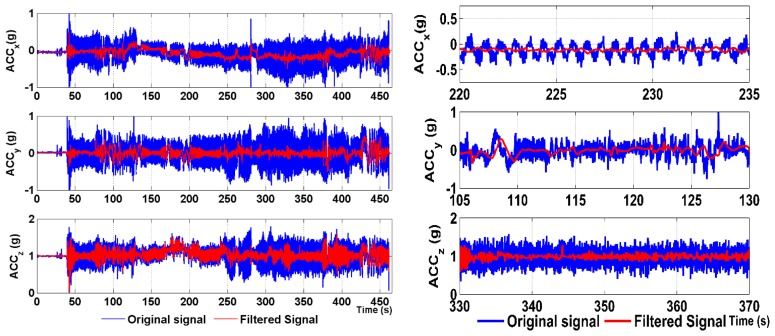
Acceleration measured during engine suppression, the filtered signal and the zoomed tracks.

Figure 10 shows the ACC signal from the same flight as shown in Figure 9.

It can be seen in Figure 10 that the low frequency vibrations affecting the signal during the engine suppression between 220–235 s are significantly attenuated in the case of *ACC_x_* (zoomed tracks). This measured signal suffered from a vibration frequency of about 0.5 Hz, and thus, at this point, the filtering was performed at the lowest bandwidth corresponding to the highest filtering order. On the other hand, the *ACC_y_* track in the range of 105–130 s suffered from high frequency vibrations; hence, the filtering was performed with a wider bandwidth; so, the filtering order was lower and the delay was shorter.

**Figure 11.**
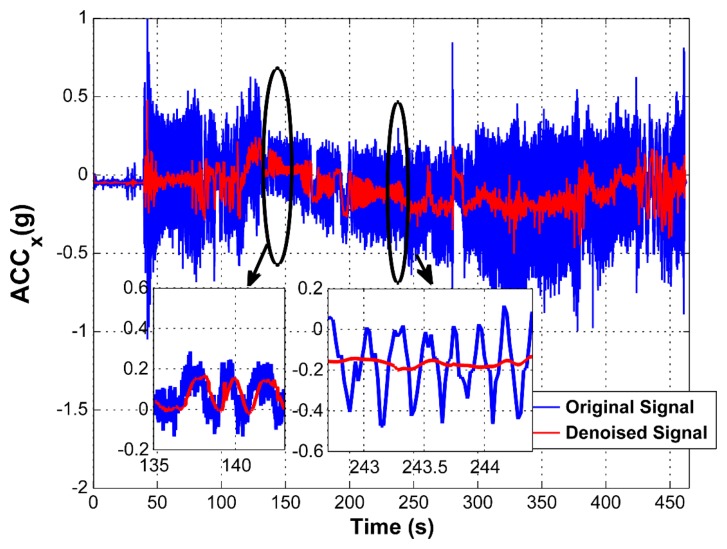
Variable bandwidth filtering of ACC in the x-axis.

To confirm the adaptability of the filtering bandwidth, the *ACC_x_* signal is shown in Figure 11 with zoomed parts. It demonstrates the variable bandwidth filtering capability and corresponding delays. In the left inset, the signal frequency was approximately 1.25 Hz and the filtering bandwidth was set to 0.5 Hz, which corresponds to the narrowest filtering bandwidth and which operates at the highest level of wavelet decomposition, *i.e.*, LoD = 3. At this instance, the order of the filter is maximal and the time delay corresponds to 0.39 s. In comparison, in the right inset, it can be seen that the signal frequency content is approximately 4 Hz and the filtering bandwidth corresponds to 3 Hz. In this instance, the time delay is 0.36 s, because the order of the filter is lower. In addition, it can be seen that a better attenuation is achieved, while the signal frequency is higher.

Nevertheless, the variable filtering of ACC signals is applied only when the character of the signal content is periodic; otherwise, a constant 5.5-Hz bandwidth filter is applied the same way as used in the ARS's signals. This approach provides the same delay on both ARS and ACC data during the majority of the flight, and when low frequency vibration content occurs in ACC data, which is generally under special and rare conditions, such as the engine RPM suppression, the bandwidth of ACC data filtering is modified. This case leads to different delays for the ARS and ACC data; however, as long as they are used only for attitude compensation under steady-state conditions, these differences can be simply managed by taking different delays in data fusion into account.

## 4. Conclusions

This paper proposed a novel concept of filtering inertial data with an enhanced capability of providing smooth data under harsh environments, eliminating low frequency vibration influences. Cost-effective attitude and heading reference systems (AHRSs) generally fuse data from angular rate sensors (ARSs) and accelerometers (ACCs) to provide stable attitude estimation. Commonly, it is advantageous to fuse data in such a way that very low-frequency content corresponding to the steady-state flight conditions is taken from the ACC's measurements and higher-frequency content corresponding to changes of flight conditions is obtained from the ARS's measurements. Nevertheless, a problem arises when ACC readings are affected by low-frequency vibrations, and thus, the compensation ability in the attitude estimation process becomes vibration dependent. This is often the case when the correct filtering is not applied on the ACC's signals. The contribution of this paper thus lies in proposing the filtering of the ACC's with an adaptive bandwidth capability, providing the same delay in inertial data processing, when vibration frequencies are above 10 Hz. When the vibration frequency in the ACC's data is below 10 Hz, the data are filtered with a modified bandwidth to reduce the vibration effects. The modification of the filtering bandwidth relies on continuous estimation of the frequency of the strongest vibration content based on the particular bandwidth filtering applied. This filtering approach was confirmed based on simulated and real-flight data, and in all cases, the proposed approach reached better efficiency for vibration impact reduction, while preserving shorter processing delays compared with the commonly used approaches used in the commercially available AHRS systems. This paper presents the data pre-processing in terms of data filtering and not data fusion. Nevertheless, based on the effectiveness of vibration impact reduction, the proposed approach improves the ACC-based attitude compensation capability even under strong vibration, which brings a significant advantage compared with the commercially available systems.

## Figures and Tables

**Table 1.** Filtering efficiency at different filtering bandwidths for various signal frequencies.

		**Signal Frequency (Hz)**																
		**14**	**12**	**10**	**8.0**	**6.0**	**5.0**	**4.5**	**4.0**	**3.5**	**3.0**	**2.5**	**2.0**	**1.5**	**1.0**	**0.6**	**0.5**	
**Filtering Bandwidth (Hz)**	**5.5 (M = 24, L = 1)**	−34.5	−31.5	−22.3	−9.0	−6.0	−0.5	0.1	0.3	0.0	−0.1	−0.3	−0.2	−0.1	−0.1	0.1	0.1	**Attenuation (dB)**
	**5.0 (M = 28, L = 1)**	−45.7	−35.2	−29.9	−24.1	−8.9	−3.7	−0.5	0.5	0.4	0.0	−0.1	−0.2	0.0	−0.1	0.0	0.0	
	**4.5 (M = 30, L = 1)**	−42.7	−38.3	−32.6	−31.0	−28.7	−6.2	−2.5	−0.6	0.0	0.1	0.0	−0.4	−0.4	−0.3	0.0	0.0	
	**4.0 (M = 26, L2)**	−42.6	−38.6	−33.7	−28.8	−24.1	−16.1	−7.4	−3.4	−0.6	0.0	0.1	0.0	−0.3	−0.4	−0.1	0.0	
	**3.5 (M = 29, L2)**	−36.7	−35.0	−32.8	−30.1	−23.4	−22.5	−15.4	−7.7	−3.7	−0.9	0.5	0.5	0.5	0.5	0.1	0.1	
	**3.0 (M = 32, L = 1)**	−53.7	−40.8	−40.8	−36.5	−29.1	−26.1	−24.9	−26.6	−9.4	−3.7	−0.3	0.5	0.3	0.1	−0.1	0.0	
	**2.5 (M = 28, L = 2)**	−45.6	−44.7	−42.3	−37.5	−29.8	−31.1	−22.8	−23.0	−21.2	−12.8	−6.0	−1.2	−0.1	0.7	0.4	0.0	
	**2.0 (M = 32, L2)**	−41.5	−38.3	−43.0	−33.8	−37.3	−29.2	−36.3	−25.9	−28.0	−19.3	−9.4	−3.6	−0.6	0.0	0.1	0.1	
	**1.5 (M = 7, L = 3)**	−40.7	−40.1	−45.6	−29.9	−41.4	−37.5	−35.5	−34.5	−21.4	−12.7	−17.1	−4.2	−2.5	−0.9	0.0	0.0	
	**1.0 (M = 38, L = 1)**	−62.7	−62.5	−61.6	−59.4	−57.5	−56.4	−58.2	−56.4	−59.4	−43.3	−26.2	−15.3	−8.2	−3.6	−0.5	0.0	
	**0.5 (M = 33, L = 3)**	−39.2	−40.1	−38.8	−38.5	−40.0	−40.1	−29.4	−37.8	−27.3	−37.6	−24.1	−35.3	−20.5	−9.8	−4.5	−3.9	

Note: M corresponds to the Kaiser windowed filter order, and L corresponds to the level of wavelet decomposition (LoD).

**Table 2.** Time delays for the chosen filtering bandwidths.

**Filtering Bandwidth (Hz)**	**Time Delay (s)**
5.5	0.23
5.0	0.24
4.5	0.26
4.0	0.33
3.5	0.35
3.0	0.36
2.5	0.37
2.0	0.38
1.5	0.38
1.0	0.39
0.5	0.39
